# Correction: Homo-condensation of acetophenones toward imidazothiones

**DOI:** 10.1039/d1ra90004h

**Published:** 2021-01-20

**Authors:** Phuc Hoang Pham, Phuc Thai Thien Nguyen, Thuy Thu Bui, Hien Nhat Tra, Tung Thanh Nguyen, Nam Thanh Son Phan

**Affiliations:** Faculty of Chemical Engineering, Ho Chi Minh City University of Technology (HCMUT) 268 Ly Thuong Kiet, District 10 Ho Chi Minh City Vietnam tungtn@hcmut.edu.vn; Vietnam National University Ho Chi Minh City Linh Trung Ward, Thu Duc District Ho Chi Minh City Vietnam

## Abstract

Correction for ‘Homo-condensation of acetophenones toward imidazothiones’ by Phuc Hoang Pham *et al.*, *RSC Adv.*, 2020, **10**, 40225–40228, DOI: 10.1039/D0RA03047C.

The authors regret the omission of two references, shown below as ref. 1(a) and (b), which should have appeared as ref. 7(a) and (b).

On page 40227, at the end of the paragraph which starts “With the results in hand, we proposed a possible mechanism for the annulation (Scheme 5)…” the following sentence should have been added:

“It should be noted that Asinger and co-workers reported a similar transformation using ammonia, instead of ammonium acetate, that occurred in methanol solvent.^1^ For that reason, an alternative mechanism should not be excluded.”

The Royal Society of Chemistry apologises for these errors and any consequent inconvenience to authors and readers.

## Supplementary Material
